# Sense and Immunity: Context-Dependent Neuro-Immune Interplay

**DOI:** 10.3389/fimmu.2017.01463

**Published:** 2017-11-03

**Authors:** Simmie L. Foster, Corey R. Seehus, Clifford J. Woolf, Sébastien Talbot

**Affiliations:** ^1^F.M. Kirby Neurobiology Center, Boston Children’s Hospital, Boston, MA, United States; ^2^Department of Neurobiology, Harvard Medical School, Boston, MA, United States; ^3^Department of Psychiatry, Harvard Medical School, Boston, MA, United States; ^4^Depression Clinical Research Program, Massachusetts General Hospital, Boston, MA, United States; ^5^Département de Pharmacologie et Physiologie, Université de Montréal, Montréal, QC, Canada

**Keywords:** allergy and immunology, sensory neurons, asthma, inflammation, neuro-immunological signaling

## Abstract

The sensory nervous and immune systems, historically considered autonomous, actually work in concert to promote host defense and tissue homeostasis. These systems interact with each other through a common language of cell surface G protein-coupled receptors and receptor tyrosine kinases as well as cytokines, growth factors, and neuropeptides. While this bidirectional communication is adaptive in many settings, helping protect from danger, it can also become maladaptive and contribute to disease pathophysiology. The fundamental logic of how, where, and when sensory neurons and immune cells contribute to either health or disease remains, however, unclear. Our lab and others’ have begun to explore how this neuro-immune reciprocal dialog contributes to physiological and pathological immune responses and sensory disorders. The cumulative results collected so far indicate that there is an important role for nociceptors (noxious stimulus detecting sensory neurons) in driving immune responses, but that this is highly context dependent. To illustrate this concept, we present our findings in a model of airway inflammation, in which nociceptors seem to have major involvement in type 2 but not type 1 adaptive immunity.

## Inflammation is a Nervous Thing

Inflammation was first characterized by Celsus as having four cardinal signs, *dolor* (pain), *calor* (heat), *rubor* (redness), and *tumor* (swelling) (1). Although we typically think of inflammation as an immune-mediated phenomenon, each of these characteristics is essentially due to neuronal activation. The local depolarization produced by the direct activation of membrane transducers in the peripheral terminals of nociceptors by noxious stimuli initiates action potentials which travel orthodromically from the periphery to the central nervous system to initiate reflexes (withdrawal, scratching, cough) and sensations (pain, itch) (2, 3). However, when the action potentials reach branch points of the sensory neurons they are also transmitted antidromically back to the peripheral terminals in a process known as the *axon reflex* (4, 5). The local depolarization and the action reflex are the means by which neurogenic inflammation is initiated. Calcium influx through voltage-gated calcium channels triggers the rapid and local release of neurotransmitters from activated peripheral terminals or those invaded by antidromic action potentials (4, 5). Neuropeptides, such as calcitonin gene-related peptide (CGRP) and substance P (SP), act on endothelial and smooth muscle cells to produce redness and heat (secondary to vasodilation) and neurogenic edema (secondary to plasma extravasation due to increased capillary permeability) (6, 7). This neurogenic component of the response to acute injury has long been recognized. However, the sensory neuronal involvement goes beyond contributing just to the vascular component of the inflammatory response to infection or injury. Complex reciprocal interactions between the sensory nervous and the immune systems have recently been detected that drive both inflammatory pain hypersensitivity and immune cell recruitment and activation (2, 8).

An early indication that nociceptors play a major role in autoimmunity was the observation that denervation of a limb following a nerve injury prevented the subsequent development of arthritis in that limb (9). This clinical finding can be recapitulated in rodents, where eliminating sensory fiber innervation decreases inflammation in models of rheumatoid arthritis (10). Although denervation can protect from arthritis, blocking nerve activity can worsen experimental inflammation, as observed in a serum-transfer model of arthritis (11). These findings seem contrary, but may indicate that nociceptors play distinct roles during different phases of the immune response in arthritis; perhaps they are required for initiation of disease, but limit arthritic effector responses (mimicked in the serum-transfer model). During initiation of immune responses, the peptides produced by and released from the peripheral terminals of sensory neurons can direct the differentiation of lymphocytes (8, 12–18) and promote the recruitment of immune cells, and therefore may determine the localization, extent, duration, and type of inflammation taking place. In consequence, this neuro-immune interaction may result in a broad spectrum of different pathophysiological changes and disease states. Understanding the specifics of the interactions between sensory neurons and immune cells and defining the rules under which they operate will open new avenues for understanding immunity and for developing novel therapeutic strategies (2). In this perspective, we highlight context-dependent aspects of neuro-immune interactions involving sensory neurons.

## Physiological Basis for Context-Dependent Neuro-Immune Communication

The primary goal of the immune system is to sense pathogens and respond accordingly for their effective removal, while limiting tissue damage and deleterious autoimmunity (19). To mount these responses, the immune system relies on an arsenal of immune cell subtypes specifically programed to eradicate a myriad of distinct pathogens. For example, different subpopulations of effector cells arise during activation of mature naïve CD4^+^ T cells by innate cells responding to distinct environmental cues, yielding highly adaptable responses to the type of pathogen. Although they lack somatically re-arranged antigen receptors, different innate lymphoid cell (ILC) lineages also contribute to differential pathogen responses (20). Historically, immune responses have been classified into three distinct groups classified by the type of T helper cell induced: type 1 responses, which provide protection against intracellular microbes through, in part, the activation of phagocytes (21); type 2 responses, which defend against parasites such as helminths (also triggered by allergens, venoms, and toxins) (22); and type 3 responses that protect against extracellular pathogens (including yeasts/fungi) (23). Type 1 immunity is characterized by T-bet^+^ IFNγ-producing ILC1, CD8^+^ cytotoxic T cells, and CD4^+^ Th1 cells which defend against intracellular virus and microbes through activation of CD8^+^ cytotoxic T cells. Type 2 immunity is defined by GATA-3^+^ ILC2, and Th2 cells that produce IL-4, IL-5, and IL-13, which induce mast cell, basophil, and eosinophil activation, as well as IgE antibody production (24). Type 3 immunity is mediated by ILC3s, and Th17 cells that produce IL-17, IL-22 (25), or both, which activate mononuclear phagocytes but also recruit neutrophils and induce epithelial antimicrobial responses.

For each of these different types of immunity, there are several stereotypical components. First, the insult must be “sensed.” In the immune system, pattern recognition receptors (PRRs) have evolved to respond to a diverse array of stimuli (26). PRRs include TLRs, C-type lectin-like receptors, and NOD-like receptors, which are expressed on the surface and in intracellular compartments of a variety of cell types, including dendritic cells (DCs) and epithelial cells. Triggering PRRs activates DCs, which then stimulate antigen-specific T cells and tune the type of Th cell response required (i.e., type 1, 2, or 3). Indirect activation of DCs can also occur, for example, *via* epithelial cell-derived factors, such as thymic stromal lymphopoietin (TSLP), which perhaps conditions DCs to induce Th2 cell responses (27). Triggering type 2 immunity may also involve DC PRRs, enzymatic activity of allergens with intrinsic protease activity, recognition of tissue damage, or distinct metabolic changes caused by allergens or helminths (22).

Next, information must travel to peripheral immune tissues, such as spleen and lymph nodes, where it is transmitted to managerial cells—mostly T helper cells, which are instructed by innate immune cells to differentiate into effector populations. Typically, primed DCs, in addition to presenting antigen in the context of costimulatory molecules to naïve T cells (Th0), will also instruct naive T cells to differentiate into an appropriate effector type (Th1, Th2, Th17, and Treg) by releasing type-specific cytokines (22). For example, in a type 1 bacterial infection, DC make IL-12, IL-6, and IL-1β which drive Th1 cell differentiation (21); (b) while in a type 17 infection, TGF-β, IL-6, and IL-23 enhance ROR-γt and STAT3 activity and drive Th17 differentiation (23); (c) in type 2 inflammation, IL-4 from DCs, epithelial cells, basophils, CD4^+^ Th2 cells, or ILC2 cells stimulates GATA-3 and STAT5, STAT6 to promote Th2 differentiation (22, 28, 29). Once polarized, the effector T cells travel back to the original location of the insult to direct appropriate host defense responses. They do so *via* the release of an “effector” sets of cytokines, such as IFN-γ by Th1 cells; IL-17, IL-21, and IL-22 by Th17 cells; and IL-4, IL-5, IL-9, and IL-13 by pro-inflammatory Th2 cells (24, 30). In disease states, these physiological responses are subverted to pathology. Each inflammatory disease is generally dominated by one particular type of immunity, which depends on the initial trigger and the individual’s micro- and macroenvironment and genetic susceptibilities. For example, types 1 and 3 immunities mediate autoimmune diseases (rheumatoid arthritis and ulcerative colitis), whereas type 2 responses cause allergies (asthma and eczema).

## Where Do Neurons Fit into This Model?

The somatosensory nervous system is positioned anatomically to be able to directly modulate immunity in secondary and primary lymphoid tissues, skin, and mucosa, by interacting with immune cells in locations where they are stationed to monitor for disturbances in barrier function and homeostasis (31–34). There is also crosstalk between the sensory neurons and the epithelial barrier (35), another important determinant of immune responses (36). In addition, sensory neurons express a wide array of ion channel receptors specialized for detecting danger signals. These danger signals can include excess heat or cold (TRPV1, TRPM8), chemical (TRPA1), and mechanical (Piezo2) insults (2, 5, 37). They can also sense protons (TRPV1) and the ATP released upon tissue damage and apoptosis (P2X3R). After neurons sense environmental cues, the acute sensory input travels to the CNS, prompting withdrawal and defensive reflexes (fever, cough, scratch, and vomiting) (2, 38–41).

Neuronal activation by the immune system has been fairly well studied. Nociceptors are activated by cytokines: IL-1β (42) or CCL3 (43) in the context of pain, IL-5 during allergic airway inflammation (8), IL-31 during lymphoma-associated itch (44), TSLP and IL-4 during atopic dermatitis (35), IL-33 after contact with poison ivy (45), and IL-23 during psoriasis (46). While TNF-α leads to the activation of nuclear factor κB (NF-κB) (47), MAP, and tyrosine kinases are typically downstream of IL-1R in sensory neurons (42). These kinases, including p38 (48), JAK1 (49, 50), and the transcription factor STAT3 (51), are just some of the downstream signaling molecules that lead to ion channel sensitization, a state of heightened activity. Much of nociceptor sensitization is related to a decrease in the activation threshold of TRPV1 or TRPA1 (52, 53), and of the sodium channels Nav1.7, Nav1.8, and Nav1.9 (54, 55), lowering nociceptor activation threshold results in pain hypersensitivity and increased itch, but also greater local release of neuropeptides that in turn activate immune cells to release more cytokines (2). Overall, inflammation leads to the simultaneous release of many pain sensitizing mediators. As a consequence, pharmacologically targeting only one of these agents may have limited effects. In addition, there is a negative feedback loop: IL-1β and IL-4 may, *via* the JAK–STAT axis, trigger increased expression of opioid receptors in neurons and, therefore, sub-acutely have anti-nociceptive and anti-inflammatory effects (56, 57).

## Pathogen Defense

We increasingly recognize that nociceptors play a much broader role in sensing their environment than originally thought; they can also directly detect bacteria and fungi, as well as may products of the immune response, including cytokines, chemokines, and immunoglobulins (2, 5, 37). Nociceptors express a variety of PRRs that directly recognize bacteria (58) and fungi (59, 60). *Staphylococcus aureus* is able to activate nociceptors through a twofold mechanism: (a) secreted pore forming toxins, for example, α-hemolysin, which permeabilizes the nociceptors’ cell membrane allowing influx of extracellular cations and (b) *N*-formylated peptides that are membrane-bound peptides that activate their cognate receptors (formyl peptide receptors) on the surface of nociceptors (58). Following experimental ablation of these neurons, the pain associated with bacterial infection is, as expected, decreased, but surprisingly local immune infiltration is increased. These data suggest that bacteria may have evolved the capacity to activate and co-opt nociceptor function to dampen innate immune responses and facilitate their survival (58). However, the neuronal response may be distinct for different types of pathogens. In the context of *Escherichia coli* peritonitis infection, transection of the vagus nerve decreased ILC3 cell numbers, reduced pro-resolving mediator levels, and altered peritoneal macrophage numbers. Exogenous acetylcholine or pro-resolving mediators restored tissue resolution tone and host responses to *E. coli* infections (61). In intestinal organ cultures, capsaicin-sensitive enteric nervous system neurons seem to distinguish among different bacterial infections (62). For example, despite belonging to the same Clostridium bacterial subset, only a few nociceptors respond to *P. magnus*, while up to 60% of the capsaicin responsive nociceptors showed calcium flux when exposed to *C. Ramosum*. Interestingly, *P. magnus* downregulates genes encoding for SP, Secretogranin III, and galanin while it upregulates neurotensin and angiotensin. Intriguingly, *C. Ramosum* had the opposite effect (62). This seems to indicate that nociceptors may be able to change their activation depending on the type of immune response elicited by commensal vs pathogen and contribute in this way to either regulatory or type1/17 immunity.

Beyond their sensory role, neurons also promote host defense and do this in part by direct interaction with immune cells. The activation of sensory neurons results in the secretion of neuropeptides from their peripheral terminals that cause the recruitment, activation, and influx of immune cells (37, 63–66). CGRP and VIP can bias DCs to produce type Th1, Th2, or Th17-skewing cytokines and enhance DCs migration to the lymph nodes (67, 68). The same neuropeptide can have multiple different effects, in some cases biasing to Th1, Th2, or serving a regulatory role (13, 67). Along with SP, these neuropeptides can also act on Langerhans (63), Th2 (68, 69), and ILC2 cells (8, 16) to change their activation states in the skin during models of contact hypersensitivity or psoriasis, and in allergic airway disease models (8). For example, in Th2-immunity, SP released by itch transducing neurons is sensed by mast cells which degranulate and release secondary mediators such as histamine, causing swelling and further enhancing the neurogenic response (3, 18, 70). Skin is innervated both by sympathetic efferent and nociceptor afferent neurons, but the denervation produced by the ablation of nociceptors alone is sufficient to reduce contact sensitivity inflammatory responses (71). Brian Kim’s group has studied the contribution of sensory neurons in AD-like skin inflammation induced by the topical irritant MC903 (calcipotriol) (50). They found that while the type 2 cytokines IL-4 and IL-13 directly activate (calcium flux) both mouse and human sensory neurons, they did not elicit acute itch. IL-4 sensitized subsets of neurons to respond to previously sub-threshold pruritogen levels (such as histamine), significantly increasing scratching. They hypothesized that, rather than acute itch, neuronal type 2 cytokine signaling promotes pathologic chronic itch and that interrupting these signals may represent an effective strategy to target itch. To test this, they generated the first sensory neuron specific cytokine receptor knockout mice (NaV1.8-Cre:IL-4R^fl/fl^) and showed that IL-4^−/−^ nociceptors mice are protected from AD-induced skin inflammation, displaying a distinct skin transcriptional profile characterized by reduced infiltration of Th2 and basophils numbers. In a distinct skin inflammatory model, toxin-induced ablation of Nav1.8^+^ nociceptors reverse imiquimod (*via* TLR7)-mediated psoriasis-like inflammation (46) while cutaneous denervation in psoriatic mice reduces the number of immune cells in lesions (72).

## Inflammatory Reflex

While the sensory neuron axon reflex contributes to local tissue immune cell recruitment and activation, immune homeostasis is also under autonomic control, in the form of an “anti-inflammatory reflex.” This systemic circuit starts when innate immune stimuli activate peripheral vagal afferents and terminates with efferent parasympathetic neurons inhibiting cytokine production by splenic macrophages, attenuating inflammation (32, 73). In the efferent arc, action potentials travel down the vagus in preganglionic motor fibers to the celiac ganglion to activate postganglionic adrenergic neurons that innervate the spleen. These neurons release norepinephrine, activating a special population of T cells that make acetylcholine (74). Acetylcholine binds to the α7nACHR expressed by splenic macrophages and inhibits their production of TNF-α (75). Harnessing the inflammatory reflex using bioelectronic devices (76), such as non-invasive vagal nerve stimulation, can help decrease the chronic inflammation found in rheumatoid arthritis patients (77) and mice with experimental inflammatory bowel disease (78). Similarly, non-invasive vagal nerve stimulation showed improved lung function in a small patient cohort by reducing exacerbations of bronchoconstriction (79).

## Airway Immunity: A Model System for Delineating Neuro-Immune Interactions

While systemic inflammation detected by the vagus perhaps leads to an anti-inflammatory reflex, the local picture in the tissue is different. The vagus nerve innervates virtually all visceral organs, and nearly 20% of afferents neurons terminate within the airways (80). Although some lumbar neurons innervate the lung epithelium (81, 82), it is estimated that up to 95% of the innervation is of vagal origin (83). The vagal sensory neurons innervating the respiratory tract are situated in two distinct ganglia, the nodose and jugular ganglion which have distinct phenotypes, embryonic origins (neural crest vs placode), anatomical projections to the respiratory tract, and brain stem, and are likely to serve distinct functions. Most of the nodose ganglion afferent fiber express markers of nociceptors, including TRP channels (TRPA1, TRPV1, and TRPM8) (84), voltage-gated sodium channels (NaV1.7, NaV1.8, and NaV1.9), voltage-gated calcium channels (CaV2.2) and mechanosensitive channels (Piezo2) (85). While these nociceptors mostly serve a defensive role by detecting chemical, mechanical, or thermal threats and initiate essential, protective airway reflexes such as cough and bronchoconstriction (86), they may also directly respond to decreases in lung compliance leading to subconscious sighs or deep inspirations (87). Physiologically, airway nociceptors can evoke both cough and neurogenic inflammation, the latter being a consequence of the axon reflex discussed above (8, 88, 89).

There are several indications that nerves and immune cells talk to each other in the lung, and this communication may have special relevance in asthma. Asthma is a chronic inflammatory disease of the airway which is caused by a combination of environmental (90) and genetic factors (91). Asthmatic patients have a denser network of sensory fibers around airways (92, 93) and a reduced threshold for neuronal activation in response to airborne irritants (94) as well as increased neuropeptide levels in these neurons (95). Collectively these features indicate excessive activity of peptidergic sensory fibers during asthma (96). T cells clustering with nerve-contacting DCs proliferate only in the airways of mice with allergic inflammation but not in the airways of negative controls (97). Eosinophils also appear to cluster around airway nerves in patients with fatal asthma and in antigen-challenged animals (98), while eosinophil-derived basic proteins enhance activation of rat pulmonary sensory neurons (99). C-fiber denervation in rats decreases the numbers of DCs in the lung and pulmonary lymphatic immune cell influx (100) while lung nociceptor stimulation with capsaicin increases both neuropeptide release and immune cell infiltration (8, 100–104). Similarly, stimulation of sensory neurons with capsaicin in subjects with active allergic rhinitis produces a reproducible and dose-dependent leukocyte influx (105), while capsaicin desensitization reduces rhinitis allergen-challenge symptoms (106). SP, CGRP, VIP, and secretin all promote eosinophil chemotaxis *in vitro* (107). SP and neurokinin A are expressed by lung nociceptor afferents and released following a broad range of different stimuli, including allergens, ozone, or inflammatory mediators. Neurokinin 1 receptor blockade decreases mononuclear cells and neutrophils (108) and eosinophils in alveoli (109) while SP (110) or capsaicin (111) drive eosinophil influx, which suggests that sensory neuron-release of the neuropeptides may drive eosinophilia in both allergic asthma and hypersensitivity pneumonitis (112). Using single-cell RNA-seq, it was recently found that the Neuromedin U receptor 1 (NMUR1) was preferentially expressed by ILC2s after alarmin (IL-25) stimulation. Neuromedin U (NMU), the ligand of NMUR1, activated ILC2s *in vitro*, and *in vivo* co-administration of NMU with IL-25 strongly amplified allergic inflammation. Despite the limited lung innervation, NMU was found only expressed in lung afferent DRG neurons, not nodose vagal neurons, and the loss of NMU–NMUR1 signaling reduced ILC2 function, altered transcriptional programs following allergen-challenge *in vivo* and prevented the development of allergic airway inflammation (113).

Neurons also play a regulatory role in allergic airway inflammation; CD11c^+^ airway mucosal DCs are in close contact with CGRP^+^ nociceptors in both rodents and human subjects (63, 114). CGRP has also been shown to have a Th2-skewing preference (68, 115) CGRP-exposed DCs seemed to enhance Th2 type immunity (115, 116), increasing IL-4 production while decreasing Th1-associated cytokines IFN-γ and IL-2 (117–119). However, despite these reports, the majority of publications on the role of CGRP on DC in the lung suggest a predominant anti-inflammatory rather than pro-inflammatory effect. Pretreating DCs reduces the activation and proliferation of antigen-specific T cells and increases the numbers of T regulatory cells (120). CGRP specifically inhibits the maturation of DCs *in vitro* while adoptive transfer of CGRP-pretreated DCs diminishes allergic airway inflammation *in vivo*, with reduced eosinophils and increased IL-10 in bronchoalveolar lavage fluid (BALF) (65).

As reviewed by Mazzone and Undem (80), the plastic nature of vagal sensory neurons in the lung typically leads to highly context-dependent responses to inflammatory cues. For example, (1) allergen-sensitization produces a vigorous increase in the number and amplitude of IL-5-medicated calcium responses in vagal nociceptors (8); (2) allergen-challenge triggers action potential firing in nodose ganglion nociceptors of allergen-sensitized guinea pig (80) and mice (121), but not in naïve animals; (3) allergen-challenge increased the excitability of A delta fibers to mechanical activation (122); (4) 24 h following an allergen-challenge, up to 25% of large neurofilament-positive nodose ganglion neurons innervating the respiratory tract start expressing SP and CGRP (93); (5) 24 h following BDNF exposure, nodose ganglion neurons innervating the trachea start express functional TRPV1 (123); and (6) allergen-challenge lowers the action potential firing threshold and increases the spiking rate of nucleus of the solitary tract (NTS) neurons, which is the brainstem structure innervated by the vagus nerve that mediates many of its sensory functions (80). The context-dependent plasticity of nociceptors may be acute, as in the case of enhanced coughing following capsaicin or bradykinin delivery to the airways (124), or may be long-lasting due to changes in the CNS similar to the central sensitization following peripheral injury (125).

## Sensory Neuron Role in Th1 vs Th2 Immunity in the Lung

Given the data indicating substantial plasticity and a diverse set of roles for sensory neurons in different inflammatory disorders in the lung, we decided to investigate the specific role of nociceptors in regulating immune cells in ovalbumin (OVA) and house dust mite models of allergic airway inflammation (8). In this study, we demonstrated an involvement of vagal sensory neurons in the murine Th2-skewed OVA model of allergic inflammation using aluminum hydroxide (AlOH) as adjuvant. This operated through a VIP–VPAC2 axis: nociceptors by releasing VIP drive VPAC2 expressing CD4^+^ and ILC2 cells to release IL-5 which, in turn, activates sensory neurons in the lung to release more VIP (8). Similar results were obtained for the house dust mite model. This indicates that nociceptors amplify adaptive immune responses in the lung in response to allergen exposure in sensitized animals, at least in the setting of Th2 immune responses. We then found that temporary local pharmacological silencing of the nociceptors is sufficient to interrupt this pro-inflammatory signaling loop, a non-immune therapeutic strategy that has immunosuppresive action. This strategy aims to temporarily silence afferents in the adult lung using large pore ion channels as a drug entry port for charged sodium channel blockers to produce targeted action potential blockade only in activated nociceptors (126, 127). These charged molecules have no action extracellularly but block sodium channels when they get into the cell. Because of their cationic charge, they cannot permeate through the membrane but are small enough to enter cells through inflammation-activated TRPV1 and TRPA1 ion channels (126, 128).

**Figure 1:**
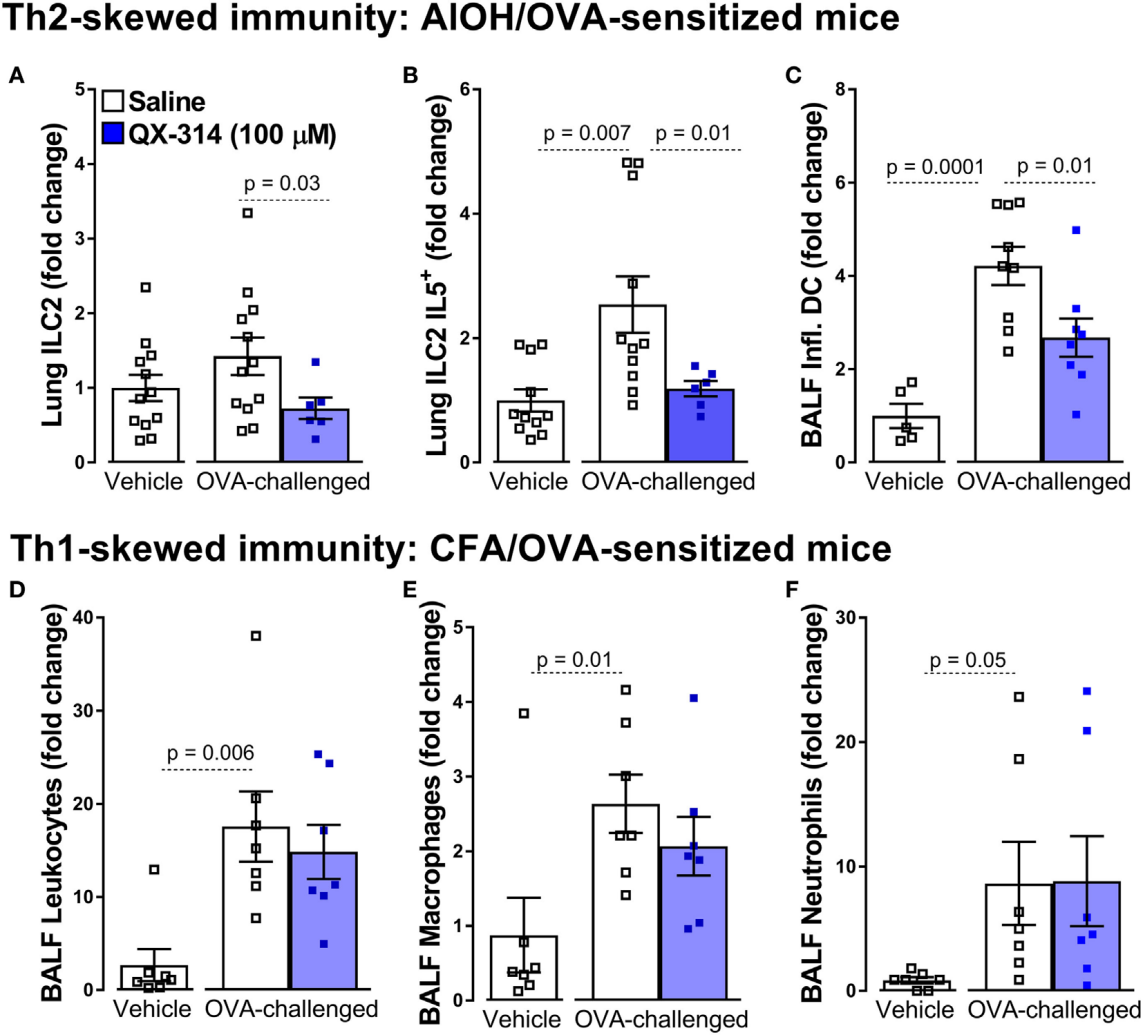
Nociceptor activation promotes Th2 but not Th1 airway inflammation. In the aluminum hydroxide (AlOH)/ovalbumin (OVA) sensitized mice **(A–C)**, a standard model of airway Th2-driven inflammation, OVA-challenge do not significantly increase the numbers of ILC2 cells **(A)** in the lung but did enhance their production of IL-5 **(B)** as well as the numbers of inflammatory dendritic cells (DCs) in bronchoalveolar lavage fluid (BALF) **(C)**. Silencing lung sensory neurons with aerosolized QX-314 (0.003%, 20 min nebulization, 20 psi) decreased these Th2 immune cell responses. By contrast, silencing nociceptors in a Th1-driven lung inflammation model [complete Freund’s adjuvant (CFA)]/OVA sensitized mice; **(D–F)**, had no impact on the OVA-challenge induced increases in BALF CD3^+^ **(D)**, eosinophils **(E)**, and macrophages **(F)**. Mean ± SEM; *Two-tailed unpaired Welsh’s t-test* (*n* = 5–12 animals/group; 1–2 cohorts).

Specifically, a single treatment with QX-314 (100 µM or 0.003%, nebulized for 20 min at 20 psi, day 18), a quaternary derivative of lidocaine, to OVA sensitized (day 0 and 7) and challenged (days 14, 15, 16, and 17) mice substantially reduces lung immune cell infiltration (day 21), with decreases in BALF numbers of leukocytes, eosinophils, macrophages, and lymphocytes (8). Building on these findings, we used this strategy to assess the influence of nociceptor ablation or silencing on the activity of primary immune drivers (ILC2 and DCs) of type 2 inflammation. Here, we generate new data showing that QX-314 abolishes the rise in lung ILC2 cells, ILC2-derived IL-5, as well as BALF inflammatory DC cells (Figures 1A–C). Thus, pharmacologic silencing of nociceptors results in decreased activation of ILC2s and Th2 effector cytokine production. The above protocol, sensitizing 8-week-old male BALB/c mice with OVA and aluminum hydroxide as adjuvant, produces a Th2-skewed inflammation (129). When mice are sensitized with a complete Freund adjuvant (CFA) and OVA, this produces non-eosinophilic Th1-skewed asthma (129). Remarkably in these mice, QX-314 treatment (100 µM or 0.003%, nebulized for 20 min at 20 psi, day 18) fails to impact the levels of CD3^+^ cells (Figure 1D), macrophages (Figure 1E), and neutrophils (Figure 1F). These data show that the contribution of *vagal nociceptors on allergic inflammation is context-dependent* with respect to the type of initial immune priming, with substantial neuronal involvement in type 2 models of allergic airway inflammation but not in a type 1 model.

## Why Would Nociceptors Participate in Th2 but Not Th1 Immunity?

Type 2 immune responses evolved to eliminate parasites and other organisms that cannot be taken care of by cell-mediated immunity. The best early defense against parasites are direct behavioral reactions elicited acutely by the parasite as a sensory-motor reflex arc; for example, the sensation of itch as a parasite invades the skin leads to a reflex action of scratching. Worms in the gut initiate peristalsis and, in the lung, parasites lead to cough and enhanced mucus production. We postulate that the neuronal response became associated with the type 2 immune response elicited by parasites in order to enable a coordinated defense response: release of histamine and IL-4 (which sensitize nociceptors), production of mucus (also due to joint neuro-immune effort), and of IgE antibodies. Thus, the linkage between sensation (airway irritancy) and behavior (cough) expanded to become a link between sensory neuron activation and immunity. Supporting this idea, allergens and other type 2 stimuli directly activate sensory neurons in tissues where parasites might be particularly active (lung mucosa, for example). An example: as discussed above, acetylcholine-producing neurons of the enteric nervous system are found in close proximity to ILC2 cells. In addition to their role in allergic inflammation, it was also recently reported that these neurons can directly sense worm products (*N. brasiliensis* excretory/secretory products) and alarmins (IL-33), and, in turn, release neuromedin U to activate ILC2 cells. Thus, ILC2-autonomous ablation of Nmur1, the NMU receptor, impaired type 2 responses and control of worm infection. These data support a role for mucosal neurons to provide immediate tissue protection against worm products and alarmins and mount type 2 inflammatory responses (130). Future experiments will determine the extent of this linkage, and whether it holds true for other organs.

## Concluding Remarks

**Figure 2:**
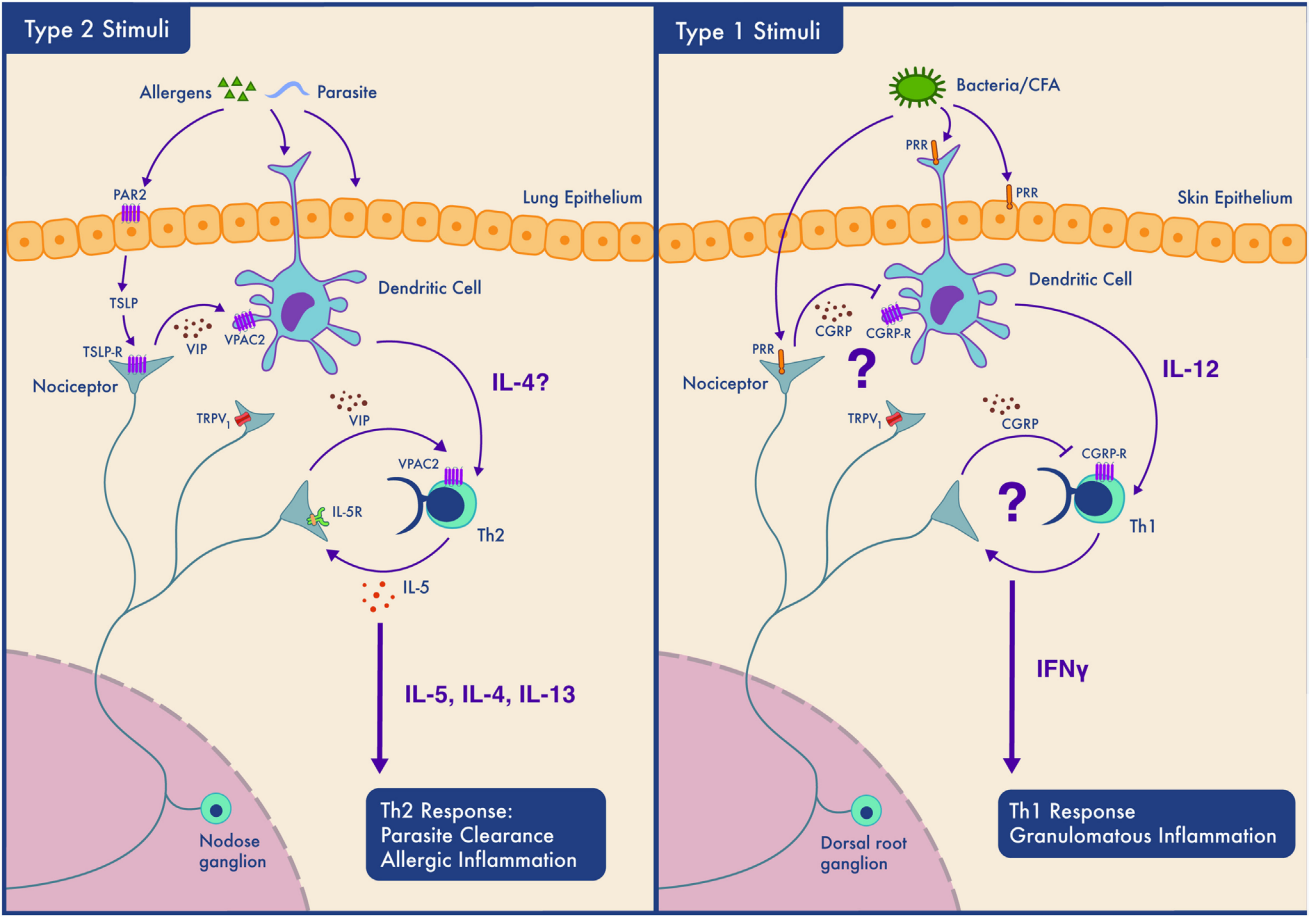
Context-dependent neuro-immune interactions; nociceptors participate in Th2 but not Th1 type inflammation depending on the tissue type. Left panel. In type 2 inflammation, allergens or parasites are sensed by epithelial and dendritic cells (DCs). Epithelial cells may secrete mediators such as thymic stromal lymphopoietin (TSLP) which sensitize nociceptors. Nociceptors release neuropeptides including VIP which act on DCs and Th2 cells and contribute to their activation. Th2 cells secrete cytokines (IL-4 and IL-5) that both drive type 2 inflammation and act on nociceptors forming an inflammatory loop. While such inflammation perhaps aids in parasite clearance by promoting coughing and mucus secretion, if amplified and prolonged, this bidirectional communication contributes to the pathology of allergic inflammation. Right panel. The case is different in our model of Th1 type inflammation where the neuropeptides secreted by nociceptors do not activate immune cells and may even actively inhibit type 1 immunity. In this situation, nociceptors may sense pathogen-associated molecules and produce neuropeptides that limit DCs activation and downstream Th1 responses.

As summarized in Figure 2, the influence of nociceptors on inflammation depends on the context (allergen sensitized or not) (80), the subtype of neuron activated, the subtype of immune cell that will respond to neuropeptide release, the location of the interaction (mucosal, epithelial, and endothelial), the timing of the interaction (acute vs chronic), and as our data suggests, the type of immune response (type 1 vs type 2). How, specifically, might nociceptors play different roles in different locations and in different types of immunity? One answer might lie in differences in expression of peptides and receptors. Nociceptors express receptors for 20 cytokines, 8 chemokines, and 6 immunoglobulins. They also express pattern recognition receptors, including two formyl peptide receptors, 11 toll-like receptors and 13 nucleotide-binding oligomerization domain-like receptors (131). Another example of the diverse functions of nociceptors is their expression of the immune checkpoint receptor PD-1 and their response to its cognate ligand (PD-L1) (132). PD-1 activation induces phosphorylation of the tyrosine phosphatase SHP-1, which inhibits sodium channels and decreases nociceptor sensitivity (132). Nociceptors also express in excess of 80 neuropeptides, each specific for receptors present at variable levels on various immune cells, creating the capacity to produce multiple different outcomes. The basal expression levels of both the neuropeptides and their cognate receptors vary in different inflammatory contexts and anatomical origins (lumbar vs vagal afferent) (131). Vagal nociceptors, for example, express higher level of Chrna5 (6.4-fold), TRPA1 (1.6-fold), VIP (16.2-fold), and IL-22R (9.2-fold) than somatic dorsal root ganglion neurons, while the latter express higher levels of KV7.5 (24.3-fold), TRPM8 (4.0-fold), CGRP (3.2-fold), and IL-31R (10.3-fold) (131).

Keeping in mind that immune cells themselves can release neurotransmitters such as dopamine by germinal center T_FH_ cells (133), we now need to examine in detail the broad repertoire by means of which sensory neurons and immune cells communicate with each other locally in damaged or infected tissue and explore where and how this contributes to disease. The development of new tools to monitor the *in vivo* activity of specific neuronal populations (by GCaMP6 and two-photon microscopy) and to stimulate (through optogenetics, DREADD), or block/ablate these neurons genetically (tetanus toxin, diphtheria toxin) or pharmacologically (QX-314), will help further dissect out the local sensory neuron–immune axis, especially when paired with single immune cell and neuron transcript profiling. Exploring the immune and nervous system contributions to inflammatory diseases will, we are confident, reveal novel therapeutic targets.

## Experimental Procedures

All procedures were approved by the Institutional Animal Care and Use Committees of Boston Children’s Hospital. Mice were housed in standard environmental conditions (12 h light/dark cycle; 23°C; food and water *ad libitum*) at facilities accredited by the Association for Assessment and Accreditation of Laboratory Animal Care. Allergic airway inflammation was studied in 8-week-old male BALB/c (stock number: 000651) mice using the classic OVA model (134) of asthma. On day 0 and 7, mice were sensitized by a 200 µl i.p. injections of a solution containing 1 mg/ml OVA (Sigma-Aldrich) and 5 mg/ml aluminum hydroxide (AlOH; Sigma-Aldrich, Boston, MA, USA). On days 14–17 (10:00 a.m.) mice were exposed to 6% OVA aerosol for 20 min. We also investigated impact of sensory neuron silencing in a TH1-skewed model of allergic airway inflammation (129) following s.c. sensitization with OVA (1 mg/ml) in a 200 µl emulsion of sterile PBS and 50% CFA on day 0 and with 50% incomplete Freund adjuvant (IFA) on day 7.

### Drugs

QX-314 (126) (Tocris) was diluted in sterile PBS to a 0.003% concentration (100 µmol) and mice were nebulized for 20 min at 20 psi on day 18 (8).

### Bronchoalveolar Lavage Fluid

On day 21, a 20 G sterile catheter was inserted longitudinally into the trachea of deeply urethane-anesthetized mice (1.5 g/kg i.p.). Two milliliters of ice-cold PBS containing protease inhibitors were injected into the lung, harvested, stored on ice, centrifuged, cells isolated, and resuspended in sterile PBS (2, 58).

### FACS

Single cells are isolated in FACS buffer (PBS, 2% FCS, EDTA), blocked (αCD16/CD32, 0.5 mg/ml, 10 min), and stained with specific monoclonal antibodies. Using a tiered gating strategy, cells are identified using light scatter parameters (FSC by SSC) and doublets are excluded. Cell populations are defined as follows: alveolar macrophages (sygF^+^CD11b^-^CD11c^+^CD64^+^), DCs (CD11c^+^CD103^+^CD24^+^FcεR1^+^), neutrophils (CD11b^+^Ly6g^+^), leukocytes (CD45^+^CD3^+^), and ILC2 (Lin^-^Thy1^+^ST2^+^CD25^Hi^) (2, 58, 135, 136).

### Cell Count

Total BAL cell counts were performed using a standard hemocytometer, with absolute cell numbers calculated as total BAL cell number multiplied by the percentage of cell subpopulation as determined by FACS (134). Data are presented as fold change in comparison to control mice.

### Intracellular Cytokine Staining

Cells were stimulated with PMA/Ionomycin in the presence of GolgiPlug (BD Biosciences) for 4 h and then fixed and stained using the BD Cytofix/Cytoperm kit following manufacturer’s instructions (BD Biosciences) (8).

## Statistics

Data expressed as mean ± SEM from 5 to 12 mice. Statistical significance determined by two-tail unpaired Welsh’s *t*-test. *p*-values less than 0.05 were considered significant. Numbers of animals are indicated on the figure.

## Author Contributions

ST and SF designed, analyzed, and performed experiment. ST, CS, SF, and CW wrote the manuscript.

## Conflict of Interest Statement

The authors declare that the research was conducted in the absence of any commercial or financial relationships that could be construed as a potential conflict of interest. The reviewer CM and handling Editor declared their shared affiliation.
